# Hydrogenolysis of Glycerol over NiCeZr Catalyst Modified with Mg, Cu, and Sn at the Surface Level

**DOI:** 10.3390/ijms25063484

**Published:** 2024-03-20

**Authors:** Norberto Vera-Hincapie, Unai Iriarte-Velasco, Jose Luis Ayastuy, Miguel Ángel Gutiérrez-Ortiz

**Affiliations:** 1 Department of Chemical Engineering, Faculty of Science and Technology, University of the Basque Country UPV/EHU, Sarriena S/N, 48940 Leioa, Spain; norberto.vera@ehu.eus (N.V.-H.); miguelangel.gutierrez@ehu.eus (M.Á.G.-O.); 2 Department of Chemical Engineering, Faculty of Pharmacy, University of the Basque Country UPV/EHU, Paseo de la Universidad, 7, 01006 Vitoria, Spain; unai.iriarte@ehu.eus

**Keywords:** ceria-zirconia, nickel catalyst, doping, glycerol, hydrogenolysis

## Abstract

Biomass valorization is an essential strategy for converting organic resources into valuable energy and chemicals, contributing to the circular economy, and reducing carbon footprints. Glycerol, a byproduct of biodiesel production, can be used as a feedstock for a variety of high-value products and can contribute to reducing the carbon footprint. This study examines the impact of surface-level modifications of Mg, Cu, and Sn on Ni-Ce-Zr catalysts for the hydrogenolysis of glycerol, with in situ generated hydrogen. The aim of this approach is to enhance the efficiency and sustainability of the biomass valorization process. However, the surface modification resulted in a decrease in the global conversion of glycerol due to the reduced availability of metal sites. The study found that valuable products, such as H_2_ and CH_4_ in the gas phase, and 1,2-PG in the liquid phase, were obtained. The majority of the liquid fraction was observed, particularly for Cu- and Sn-doped catalysts, which was attributed to their increased acidity. The primary selectivity was towards the cleavage of the C–O bond. Post-reaction characterizations revealed that the primary causes of deactivation was leaching, which was reduced by the inclusion of Cu and Sn. These findings demonstrate the potential of Cu- and Sn-modified Ni-Ce-Zr catalysts to provide a sustainable pathway for converting glycerol into value-added chemicals.

## 1. Introduction

The demand for sustainable solutions to address environmental challenges posed by growing human populations, rapid industrialization, and excessive fossil fuel consumption has increased. Biomass valorization is a crucial strategy for converting organic resources into valuable energy and chemicals, contributing to the circular economy, and reducing carbon footprints.

Biomass valorization is the process of converting biomass into high-value energy and chemicals. This can be achieved through various methods, including thermochemical processes like gasification and pyrolysis [1,2], chemical processes such as hydrolysis [3], and biological methods like anaerobic digestion [4]. Each method has its own advantages and challenges. For example, thermochemical processes can be expensive, while biological methods may have lower yields. The integration of thermochemical and biological processes offers a promising solution to the challenges mentioned [5]. This enhances resource efficiency and reduces environmental impact. Integrated biorefineries, which combine various conversion technologies, mark a crucial step towards sustainable bioenergy and bioproduct production, including biofuels such as biodiesel [6,7].

Biodiesel production is a sustainable alternative to fossil fuels that also generates glycerol, a byproduct with significant potential in sustainable chemistry due to its versatile properties that makes it an ideal raw material for a wide range of products. The valorization routes for glycerol depend on the activation and reactivity of specific bonds such as C–C, C–O, C–H, and O–H. The preferential cleavage of these bonds determines the catalyst properties and process conditions [8,9]. Bifunctional catalysts containing both metal and acid/basic sites are suggested as the most suitable for cleaving C–C and C–O bonds [10].

Several methods have been investigated for converting glycerol into fuel alternatives using technologies developed for glycerol valorization. Glycerol steam reforming, which uses nickel-based and noble metal catalysts, has attracted significant interest due to the high concentration of hydrogen it produces [11,12,13]. At the same time, liquid-phase hydrogenolysis has emerged as another important approach [14]. This method uses hydrogen, which is often produced via reforming processes, to convert glycerol into a variety of valuable chemicals. Hydrogenolysis is particularly attractive because it operates under milder conditions (around 200 °C and 30 bar) [15]. This makes it an excellent option for glycerol valorization [16]. Coupling the hydrogenolysis with the aqueous phase reforming (APR) of glycerol produces H_2_ in situ, making it a more sustainable and safer alternative to traditional methods that require the external addition of H_2_. The APR and hydrogenolysis can be easily integrated into a one-pot reaction due to their comparable operational conditions. During this process, notable reactions occur, including dehydration–hydrogenation, which facilitates hydroxyacetone formation at Lewis acid sites, and the dehydrogenation–dehydration–hydrogenation sequence, which is particularly effective under basic conditions [17,18]. These pathways are crucial for the production of propylene glycols (PG), particularly 1,2-PG, due to its widespread use as a platform molecule in the industry.

Bimetallic catalysts have demonstrated promising outcomes in glycerol hydrogenolysis [19,20,21]. The addition of promoters such as Sn, Mg, Co, and Cu enhances selectivity through various mechanisms, such as blocking surface defects and reducing particle size [22]. Ni-Mg catalysts exhibit increased H_2_ selectivity by enhancing nickel’s electronegativity, reducing the dehydration pathway [23]. Ni-Cu catalysts were found to have better Ni dispersion, reducibility, and WGS, while also resisting oxidation, sintering, and coke formation [24]. On the other hand, Ni-Sn catalysts were found to suppress CO methanation by modifying nickel’s defective sites, offering increased sintering resistance [25].

This study investigates the effect of Mg, Cu, and Sn on the performance of NiCeZr catalysts in glycerol hydrogenolysis. The undoped NiCeZr catalyst has been proven effective for producing valuable liquids through glycerol hydrogenolysis [26]. In this study, we analyze the structural and surface changes that occur upon the addition of dopants and evaluate their effect on reaction efficiency and selectivity. The catalysts synthesized were characterized in both their fresh and reduced forms to establish a correlation between their physicochemical properties and catalytic performance. Additionally, the spent catalysts were characterized to identify the primary causes of deactivation.

## 2. Results and Discussion

### 2.1. Textural Properties and Bulk Chemical Composition

The NiCeZr base contained 9.2 wt.% of Ni and a Ce/Zr atom ratio of 85.9/14.1, which is close to the targeted values. The promoters were loaded according to the targeted values, except for Sn, which had a 20% deficit (Table 1). All the solids exhibited a type IV (a) isotherm with an H_2_ (b) hysteresis cycle (Figure S1, Supplementary Materials); these are characteristics of mesoporous solids with a bottleneck pore structure. Cu and Sn impregnation blocked the smallest pores, resulting in a slight increase in the average pore size compared to NiCeZr (Table S1, Supplementary Materials). However, the impregnation of Mg had little effect on the pore sizes.

The calcined NiCeZr assay had a S_BET_ of 235 m^2^/g and decreased by 4–15% (depending on the modifier) after impregnation (Table 1). After impregnation with Mg and Cu, the pore volume tended to increase, likely due to the formation of larger pores, while impregnation with Sn reduced the V_pore_. The surface tension of the water and isopropanol mixture used to impregnate Sn was lower than that of water alone. This could facilitate greater wetting and allow Sn to penetrate all pores more effectively, thereby decreasing pore volume.

The pore structure of the reduced solids remained unchanged since the isotherms showed little variation. The average pore size of Mg/NiCeZr and Cu/NiCeZr solids increased slightly compared to their calcined counterparts. The catalyst impregnated with Sn exhibited bimodal PSD curves. The S_BET_ of the reduced solids decreased if compared to their calcined counterparts, (by 10–19%) except for Sn/NiCeZr, which was fully retained. Interestingly, all the modified solids exhibited a greater S_BET_ than the base NiCeZr, suggesting that promoters enhanced the thermal stability of the solids.

### 2.2. Structural Characterization

**Figure 1:**
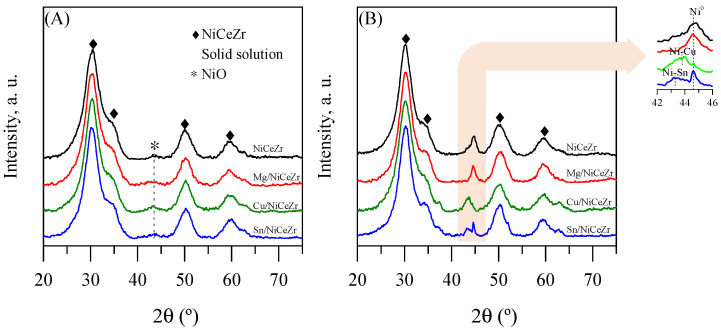
XRD profiles of the (**A**) calcined, (**B**) reduced solids.

The XRD diffractograms of the calcined solids (Figure 1A) showed broad diffraction peaks, indicative of a small crystallite size. The most intense signals were attributed to the NiCeZr solid solution that crystallized in the tetragonal phase (ICDD 01-088-2398). Additionally, a barely discernible peak at 44.3°, attributed to NiO (ICDD 01-078-0643), was observed, suggesting that some of the nickel had segregated as NiO. After impregnating the promoters, no distinct peaks for MgO, CuO, or SnO_X_ were observed, indicating their high dispersion. These results were expected since the nominal loading of the promoters was about 1/20 of the monolayer.

Upon reduction, the dominant peaks observed in their calcined forms were preserved, with the absence of other peaks from the Ce-containing or Zr-containing phases, suggesting the CeZr mixed oxide remained. For the base NiCeZr assay, the peak from NiO disappeared and a new peak emerged at around 44.5°, which was attributable to the metallic Ni (ICDD 01-087-0712). This suggests that the nickel was reduced and remained as metallic clusters on the catalyst’s surface. A similar outcome was obtained for the Mg/NiCeZr material. The spectrum of the Cu/NiCeZr sample did not show the NiO signal. Instead, a new peak appeared at 43.7°, which was attributed to an intermetallic Ni-Cu alloy [27], together with a peak from metallic nickel (inset in the figure). The diffractogram of the reduced Sn/NiCeZr solid showed peaks from metallic nickel and NiO, the presence of the latter suggesting a lower reducibility of this solid. Thus, it could be concluded that, after reduction, nickel in different oxidation states coexisted within the Sn/NiCeZr solid, which was a similar outcome to previous studies [28].

The crystallographic parameters in Table 1 showed that the calcined materials had crystallite dimensions ranging from 2.3 to 2.8 nm. This small size was consistent with the broad diffraction peaks of low intensity in the XRD diffractograms and the high S_BET_. The solid particles were bigger than crystallites, indicating that particles formed by an aggregation of up to two crystallites (Table 1). The lattice length of the modified solids increased along the a-axis while decreasing along the c-axis, indicating structural changes upon the addition of the modifier.

After reduction, all samples showed an increase in both the dimensions of the NiCeZr crystallites and the lattice length along the a-axis. This expansion of the crystalline network could be attributed to the release of oxygen from the structure and the decrease in the oxidation state of specific cations. The repositioning of the cations and oxygen vacancies facilitated these structural changes [29]. The increase in the a-axis was found to be correlated with the atomic size of the modifier, following the sequence NiCeZr < Mg/NiCeZr ≈ Cu/NiCeZr < Sn/NiCeZr.

The size of the metallic nickel crystallites in the NiCeZr base catalyst was 11.4 nm and varied unevenly with the modifier. The most significant variation occurred in the Cu/NiCeZr catalyst (decreased to 5.0 nm) and the Sn/NiCeZr catalyst (increased to 20.1 nm), while the Mg/NiCeZr catalyst preserved the size of the Ni^0^ crystallites. These results suggest that a strong interaction between Ni and Cu led to an alloy with a reduced crystallite size.

**Figure 2:**
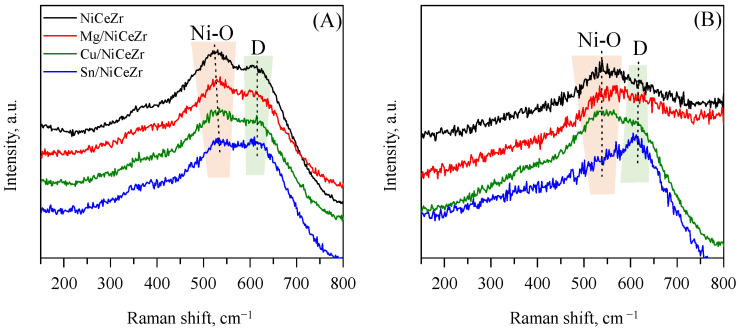
Raman spectra for (**A**) calcined and (**B**) reduced forms of the Me/NiCeZr materials.

Further characterization of the solid structures was carried out through Raman spectroscopy (Figure 2). This technique, complementing the XRD technique, is particularly useful in the analysis of NiCeZr mixed oxides [30]. The spectra of the calcined solids presented broad bands, which posed a challenge for their assignment. The broadness of the bands was caused by both the small grain size, as observed from the XRD results, and the presence of ordered defects such as anion vacancies and substituted cations [30]. For the base NiCeZr calcined solid, two primary signals were observed: one at approximately 525 cm^−1^, attributed to the Ni-O bonds, and another at approximately 615 cm^−1^, named the D band of defects, which was related to external defects resulting from the addition of Ni in CeZr solids [31]. In the surface-modified solids, a redshift of the Ni-O band was observed, suggesting alterations in the Ni neighborhood. This phenomenon was noted across all materials, indicating modifications in the electronic structure and potentially in the arrangement of atoms adjacent to Ni.

After reduction, the Raman profiles changed for all the catalysts. For example, for the base NiCeZr and Mg/NiCeZr catalysts, the D band vanished and the band from Ni-O bonds shifted to 550 cm^−1^. For the Cu/NiCeZr catalyst, the Ni-O band notably broadened, while it almost vanished for the Sn/NiCeZr catalyst. These solids were the only solids where the D band remained.

### 2.3. Redox Properties from the H_2_-TPR Analysis and Oxygen Storage Capacity

**Figure 3:**
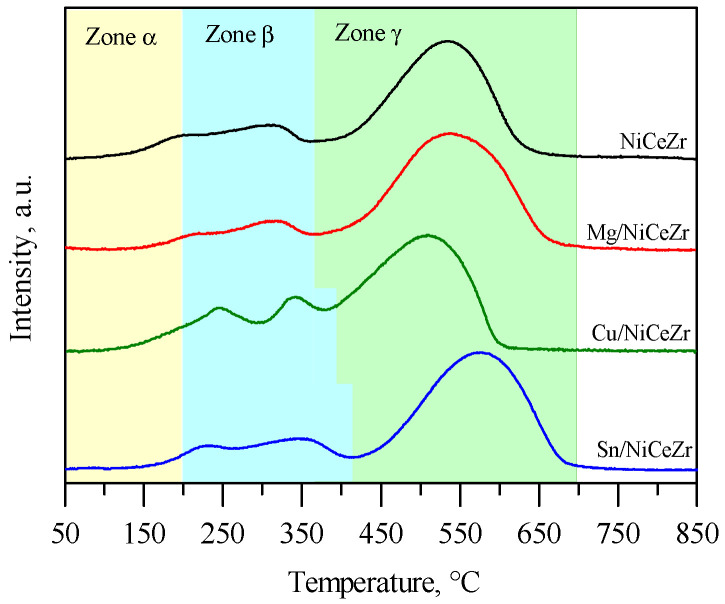
Reduction profiles of Me/NiCeZr solids.

The reduction profiles of the base NiCeZr solid (Figure 3) contained three primary contributions, each corresponding to the reduction of different chemical species [32,33]. The contribution below 200 °C (named α-contribution) was associated with the reduction of easily reducible oxygen at oxygen vacancy sites (O_V_). The intermediate temperature contribution (the β-contribution) in the range between 200 and 360 °C involved the reduction of free NiO entities on the surface. Lastly, the γ-contribution, encompassing the range from the end of the β-contribution up to 800 °C, included the reduction of Ni^2+^ species in the solid solution, as well as of Ce^4+^ ions on the surface and in bulk. The reduction profile of the Mg/NiCeZr solid was similar to that of NiCeZr, the γ-peak getting broader and upshifting by 7 °C. These results suggested that the presence of Mg somewhat hindered the reduction of Ce^4+^ and/or Ni^2+^ in the ternary solution [34,35]. The reduction profile of the Cu/NiCeZr solid differed from that of the base NiCeZr in two main ways. Firstly, there were two well-defined peaks in the β-region, corresponding to the separated reduction of highly CuO to Cu^0^ (peaking at 245 °C) and NiO to Ni^0^ (peaking at 342 °C) [36,37].

Secondly, the γ-contribution was shifted downwards from 531 °C to 505 °C, suggesting that the interactions between the copper oxide and NiCeZr support enhances the reducibility of ceria and Ni^2+^ in the solid solution [37], due to the spillover effect on metallic Cu [38]. Finally, the reduction profile of Sn/NiCeZr was similar to that of the base NiCeZr. The profile showed a general upshift across the entire range, specifically by 30–45 °C for the β- and γ-contributions. This suggests that the presence of surface SnO_2_ hindered the reduction of surface NiO, and Ni^2+^ and Ce^4+^ in the solid solution [39]. The reduction profile of the supported SnO_2_ typically exhibits two contributions in the 400–600 °C range. These contributions are attributed to a sequential Sn^4+^ → Sn^2+^ → Sn^0^ reduction process [40]. In our sample, they were masked by the intense peak from the γ-contribution.

Table 2 shows that the reduction percentage for H_2_-TPR was slightly above 100% for all catalysts except for the Mg/NiCeZr assay, which was 78%. This might be due to the decrease in available NiO. The interaction between Ni^2+^ and Mg^2+^ ions can affect the distribution of Ni^2+^ ions on the surface and subsurface of the material, with the insertion of Ni^2+^ in the MgO lattice, making it much less reducible [41].

The Mg/NiCeZr and Sn/NiCeZr catalysts showed a decrease in hydrogen consumption related to both O_V_ (α-contribution) and β-contribution. The former may be attributed to the fact that O_V_ sites could serve as anchor points for these metal oxides [42]. However, the α-contribution in the Cu/NiCeZr catalyst was similar to that of the base catalyst, as new oxygen vacancies could be formed upon Cu impregnation [43]. The catalyst showed an increase in hydrogen consumption in the beta zone by 0.18 mmol_H2_/g compared to the base NiCeZr material. This increase was attributed to the concomitant reduction of CuO entities in this temperature region. In the Sn/NiCeZr solid, there was a slight increase in hydrogen uptake in the β- and γ-peaks, which was attributed to the reduction of SnO_2_ to metallic Sn.

In summary, the addition of Cu promoted the reducibility of Ni in NiCeZr catalysts, resulting in a lower temperature of the γ-contribution. However, Mg seems to hinder the reduction of both Ce^4+^ and Ni^2+^, possibly by altering the Ni^2+^ distribution and reducibility. The presence of Sn increases the reduction temperatures, suggesting a possible hindrance in the reduction pathways of surface NiO, Ni^2+^ in the lattice, and Ce^4+^. Each promoter clearly affects the reduction profile of the catalyst.

The OSC of the base NiCeZr was assessed by the O_2_/CO pulse injection method at 235 °C and accounted for 63 mmol_O_/g_cat_, as shown in Table 2. This value was attributed to the Ce^3+^/Ce^4+^ and oxygen vacancies of the catalyst [44]. The OSC varied little upon the addition of Mg and Cu, but notably decreased by 32% upon impregnating Sn, in accordance with the hindered reducibility of this sample.

### 2.4. Characterization of Acid and Metallic Sites

An in-depth analysis was conducted to characterize both metallic and acid sites in the Me/NiCeZr catalysts. The density of the acid sites was significantly influenced by the promoter (Table 2). Specifically, the acid sites’ density of the catalyst modified with MgO decreased by 17% with respect to the undoped solid, underscoring the basic nature of alkaline earth metals [35]. Conversely, the catalysts modified with Cu and Sn notably increased their surface acid sites’ density (increases by 17% and 42% for Cu/NiCeZr and Sn/NiCeZr, respectively), in agreement with the acidic characteristics of these modifiers [45,46].

The desorption profiles of ammonia in Figure S2 (Supplementary Materials) indicate a strong effect of the modifier. The profiles consist of various peaks at different temperatures, which are indicative of acid sites of different strengths. The assignment of the peaks to their respective strengths was performed according to [26]. The contribution of each acid-strength site is summarized in Table S2 (Supplementary Materials). The impregnation of Me primarily resulted in a slight increase in the contribution of intermediate strength acid sites and a concomitant decrease in strong sites. The type of dopant, however, does not significantly affect the percentage contribution of each center. In these types of solids, acidity originates from the Lewis type, with coordinatively unsaturated cations on their surfaces [47].

The density of metallic sites was determined through hydrogen chemisorption. The results presented in Table 2 show a decreasing trend in metal site density when NiCeZr was doped with various elements. The undoped NiCeZr catalyst had 0.22 metallic sites per nm^2^. After being doped with Mg, the density of metal sites decreased by 32%, which is consistent with the H_2_-TPR results and in agreement with other studies [48]. Doping with Cu and Sn resulted in a significant decrease in metallic site density (around 90%). In the case of the Cu/NiCeZr assay, the decrease was attributed to the formation of the NiCu alloy on the catalyst surface, as observed from the XRD results [49,50]. For Sn/NiCeZr, the observed effect could be attributed to the growth of the metallic nickel crystallite (doubled with respect NiCeZr) and the presence of SnO_X_, which is inert for H_2_ chemisorption, covering the metallic nickel, as observed through H_2_-TPR.

### 2.5. Surface Characterization Using XPS Analysis

Distinctive features of both Ce^3+^ and Ce^4+^ were identified in the Ce 3d core-level spectra of all calcined samples (Figure S3A, Supplementary Materials). Two doublets labeled as v′/u′ and v0/u0 were observed for Ce^3+^, while three doublets named v/u, v″/u″, and v′′′/u′′′ were detected for Ce^4+^, corresponding to the Ce 3d_5/2_ and Ce 3d_3/2_ levels, with an energy separation of 18.3–18.6 eV between both signals, in agreement with the literature [51]. Ce^4+^ was found to be the predominant form of cerium for all the catalysts (Table 3).

**Figure 4:**
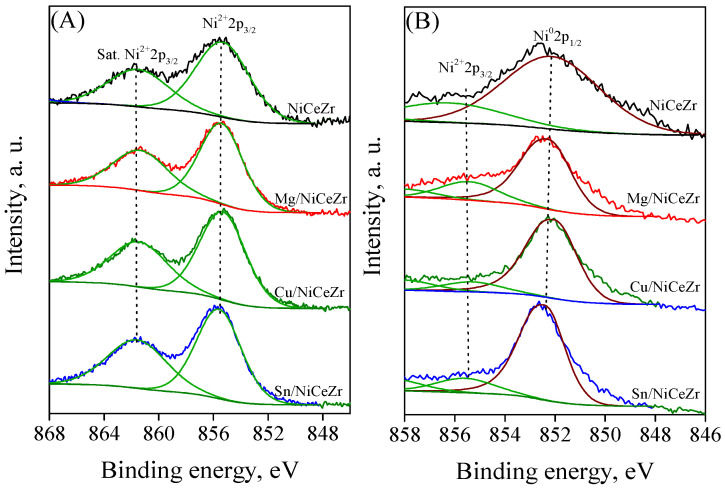
Detailed Ni 2p spectra for calcined (**A**) and reduced (**B**) catalysts.

The analysis of the Ni 2p_3/2_ core-level spectra (Figure 4) revealed a consistent pattern, consisting of a main peak in the range of 851–859 eV and a satellite peak at around 862 eV, both indicative of the Ni^2+^ species [52]. These results confirmed that the calcined solids contained only Ni^2+^. The spectrum of Mg/NiCeZr was found to be identical to that of NiCeZr. However, the main peak shifted slightly towards a lower binding energy (−0.1 eV) for the Cu/NiCeZr catalyst. This shift could be associated with changes in the unfilled electronic holes of the D band, resulting from the charge transfer from Cu to the adjacent Ni [53]. On the other hand, the main peak shifted up by 0.2 eV for the Sn/NiCeZr catalyst, which was likely due to the higher electronegativity of Sn (1.96) compared to that of nickel (1.91) [54].

The Zr 3d core-level spectra for all calcined solids (Figure S4 in the Supplementary Materials) presented a doublet at approximately 183.05 eV and 185.5 eV and corresponded to the Zr 3d spin-orbit splitting components (Zr 3d_5/2_ and Zr 3d_3/2_) of Zr^4+^ [55,56]. Upon the impregnation of Me, the Zr 3d peaks upshifted in their BE, indicating that the chemical nature of the Zr^4+^ species in these materials differed from that in the parent NiCeZr [57]. Among the three dopants used, Sn caused the largest shift, suggesting that the strongest interaction occurs between Zr and Me.

The O 1s spectrum of the calcined catalysts exhibited three distinct peaks (Figure S5, Supplementary Materials). The peak located at around 529.5 eV corresponded to the oxygen in the lattice (O_L_); the peak at 531.0 eV was linked to oxygen species adsorbed on the vacancies (O_V_); and the peak at 533 eV could be attributed to chemisorbed species, such as water, carbonate, or OH compounds (O_ads_) [58]. Ce-containing materials often have Ce^3+^ ions associated with surface oxygen vacancies, which affect the oxygen storage capacity (OSC) [59]. Our catalysts followed this linear correlation (Figure S6, Supplementary Materials).

**Figure 5:**
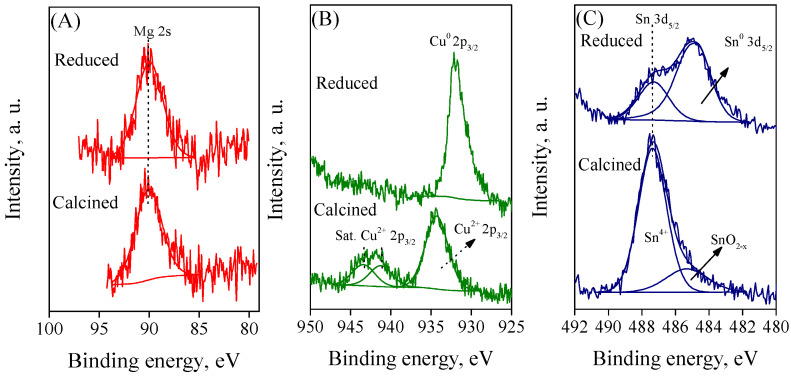
XPS spectra of Mg 2s (**A**), Cu 2p_3/2_ (**B**), and Sn 3d_5/2_ (**C**) in the calcined and reduced forms of Me/NiCeZr catalysts.

Figure 5 displays the Mg 2s, Cu 2p_3/2_, and Sn 3d_5/2_ features of calcined Me/NiCeZr catalysts. The Mg 2s core level peaked at 90.2 eV, which is slightly higher than that of MgO [60]. This could be attributed to Mg^2+^ species interacting with their surroundings, which is in agreement with H_2_-TPR. The Cu 2p_3/2_ spectrum of Cu/NiCeZr consisted of a main peak at 934.2 eV, accompanied by intense shake-up satellite peaks in the range of 939–946 eV. Both features show Cu^2+^ species [61]. The BE of the main peak in the Cu/NiCeZr catalyst was slightly higher than that of standard CuO [62], indicating a strong interaction between Cu and its surroundings. The absence of a secondary peak near 932.8 eV, typically associated with low-valence copper species such as Cu^1+^, suggests that the copper species on the surface were only present as Cu^2+^ [63]. The Sn 3d_5/2_ spectrum of the calcined Sn/NiCeZr catalyst showed a major contribution at 487.4 eV, corresponding to Sn^4+^ species, and an additional weak peak at 485.2 eV, corresponding to less oxidized tin species. The vacuum in the XPS chamber likely generated the formation of partially reduced tin. The Sn 3d_5/2_ peak of the Sn/NiCeZr catalyst shifted slightly upwards compared to standard SnO_2_ [64]. This indicates a strong interaction between Sn and its surroundings, which is consistent with the XRD and H_2_-TPR results.

For most of the elements, the core-level profiles of the reduced catalysts were notably different from those of the calcined catalysts. For example, the Ce 3d spectra of all catalysts (Figure S3B, Supplementary Materials) only showed Ce^3+^ features, indicating a complete reduction of Ce^4+^ ions to Ce^3+^, consistent with the H_2_-TPR results. Upon reduction, the Ni 2p spectra of the NiCeZr catalyst (Figure 4B) showed a major peak at 852.5 eV, indicating the presence of metallic Ni [65]. The presence of remnant Ni^2+^ in the form of the NiCeZr solid solution, with hindered reducibility, is indicated by the weak peak at around 857 eV. The intensity of the Ni^0^ peak was considerably higher than that of Ni^2+^, indicating a significant surface nickel reduction. Similar profiles were obtained for the reduced Me/NiCeZr catalysts after reduction. The intensity of the Ni^2+^ peak varied among the catalysts. It increased for the Mg and Sn-doped catalysts and decreased for the Cu/NiCeZr catalyst, which is consistent with the H_2_-TPR results. The Zr 3d signal in the reduced samples remained largely unchanged, reflecting its hard reducibility. The O 1s spectra (Figure S5B, Supplementary Materials) exhibited two changes upon reduction. The oxygen binding energy experienced a downshift of 0.2–0.4 eV, indicating the polarization of oxygen–metal bonds. Furthermore, the peak area of O_ads_ decreased, which may be attributed to the desorption of chemisorbed species resulting from the reduction treatment.

The spectrum of Mg 2s in the calcined and reduced Mg/NiCeZr catalysts was identical, indicating that MgO was not reduced under the experimental conditions. The Cu 2p_3/2_ spectrum of the reduced Cu/NiCeZr catalyst indicates the disappearance of signals corresponding to Cu^2+^ and the appearance of a new signal at 932 eV, which is associated with Cu^0^ [66]. The Sn 3d_5/2_ spectrum of the Sn/NiCeZr catalyst after reduction showed two distinct peaks at approximately 484.2 and 487.1 eV. These peaks were identified as metallic Sn^0^ and Sn^δ+^, respectively [64]. Based on the larger area of metallic Sn compared to oxidized Sn, it can be inferred that the majority of Sn was in a metallic state. The persistent presence of the Sn^δ+^ signal was attributed to incomplete reduction of SnO_2_ at 600 °C.

In addition to oxidation state information obtained from BEs, the relative surface concentration of elements can be calculated using their signal intensities. Table 3 shows that the surface of the reference NiCeZr catalyst was enriched in Zr in the calcined forms (bulk Ce/Zr: 0.164), which is consistent with the literature [67]. The study found that the surface enrichment in Zr was lower for the doped solids. The results suggest that doped metals substituted Zr on the surface, which is consistent with the increase in the *a*-axis lattice length observed from the XRD results. The surface atomic ratio of Ni/(Ce + Zr) in the calcined NiCeZr solid was lower than that in the bulk (0.232). However, the addition of a promoter (Mg, Cu, or Sn) increased the ratio, surpassing the bulk composition. The surface concentration of Ce^3+^ increased with the addition of a promoter. A charge imbalance occurred, leading to the formation of oxygen vacancies, structural defects, and unsaturated chemical bonds on the catalyst’s surface [68]. The surface impregnation method used for its addition resulted in the atomic content of each promoter on the surface being two to three times that of the bulk, as expected.

Upon reduction, all surface cerium was reduced in accordance with the literature [69,70]. Additionally, the Ce/Zr ratio on the surface increased, which is consistent with the literature [71]. However, all catalysts exhibited a decrease in the Ni/(Ce + Zr) ratio, which was attributed to the decoration of Ni particles by cerium [47]. In the reduced materials, the atomic content of each promoter on the surface was preserved.

### 2.6. Hydrogenolysis of Glycerol

**Figure 6:**
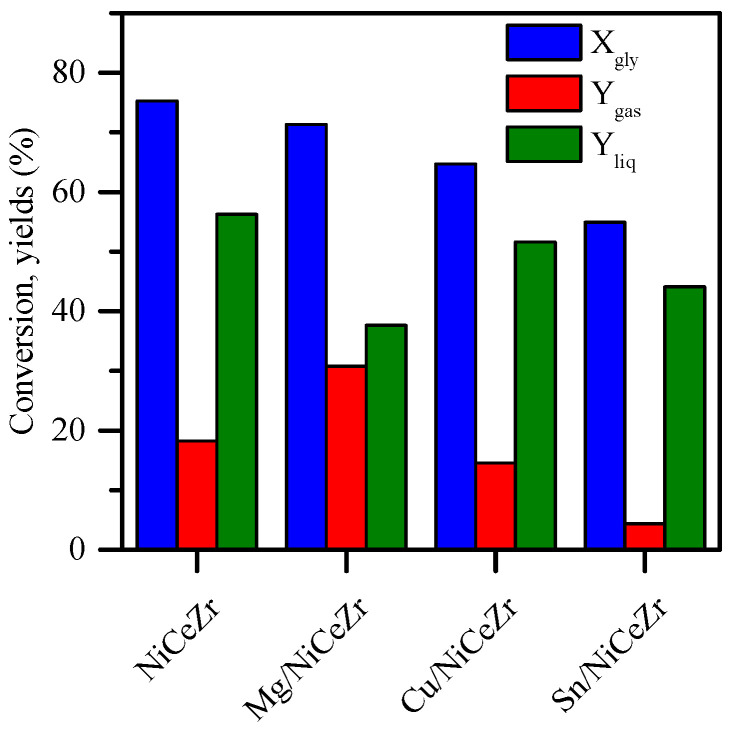
Effect of the composition of the catalyst on overall glycerol conversion and yields of liquid and gas. Reaction conditions: T = 235 °C, P = 35 bar, WHSV = 12 h^−1^, 10 wt.% glycerol, data at 3 h TOS.

The performance of the synthesized Me/NiCeZr catalysts was evaluated for glycerol hydrogenolysis in the aqueous phase without the addition of external hydrogen. The presence of gas-phase H_2_ or hydrogenolysis products would imply the production of H_2_ by sacrificing part of the fed glycerol. The NiCeZr catalyst achieved the highest glycerol conversion rate (Figure 6), with most of the reacted carbon obtained in the liquid phase (Y_liq_ > Y_gas_). The addition of any promoter resulted in lower glycerol conversion and reduced liquid yield. The type of promoter had a significant but distinct impact on both outputs. For instance, the X_gly_ decreased as follows: NiCeZr (75.3%) > Mg/NiCeZr (71.3%) > Cu/NiCeZr (64.7%) > Sn/NiCeZr (54.9%). However, Y_liq_ followed the trend: NiCeZr (56.3%) > Cu/NiCeZr (51.6%) > Sn/NiCeZr (44.1%) > Mg/NiCeZr (37.6%). There was a clear correlation between the density of the acid sites (Table 2) and the percentage of Y_liq_ in relation to the total carbon yield (Y_liq_ + Y_gas_), irrespective of the glycerol conversion, in line with others [72].

Mg increased the gas yield (30.8%), though with a corresponding decrease in glycerol conversion and liquid yield. It was found that the addition of MgO, by providing basic sites and altering the electronegativity of Ni in the catalyst, favors hydrogen formation and reduces the likelihood of feed dehydration, translating into improved selectivity towards gas production rather than liquid products [73,74,75].

**Scheme 1:**
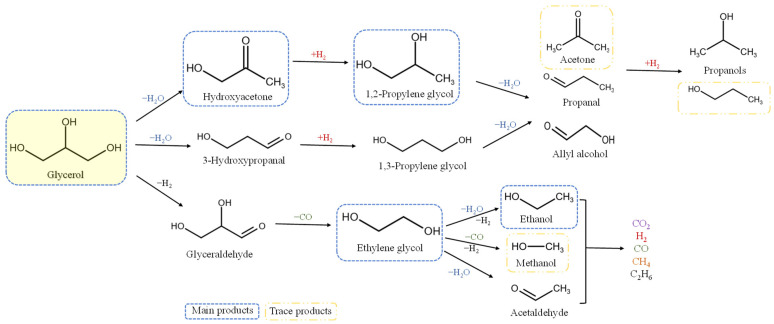
Reaction routes in the hydrogenolysis of glycerol.

The reaction scheme for glycerol hydrogenolysis using metal–acid bifunctional catalysts involves three primary pathways: dehydration, dehydrogenation, and hydrogenation reactions [76] (Scheme 1). Hydroxyacetone is formed when the primary OH is cleaved, leading to the cleavage of the C–O bond and the creation of a C=O bond (Path A). Alternatively, 3-hydroxypropanal is formed when the secondary OH is cleaved (Path B). Alternatively, glyceraldehyde is formed by breaking the O–H bond (Path C). Path A involves dehydrating the terminal hydroxyl on acid sites to hydroxyacetone (HA). Subsequent hydrogenation on metal sites produces 1,2-propylene glycol (1,2-PG). Further dehydration and hydrogenation steps can produce mono-alcohols such as 1-propanol and 2-propanol. Path B starts with the dehydration of the secondary hydroxyl to 3-hydroxypropanal, which then undergoes hydrogenation to form 1,3-PG. This pathway is preferred on Brönsted acid sites, while Lewis acid sites tend to lead to the path A products. Finally, Path C involves secondary C-C breaking reactions that are responsible for the formation of C2 and C1 products. Path C consists of sequential dehydrogenation and decarbonylation (C–C scission) on metal sites, which produces CO and ethylene glycol (EG) [77]. The pathway could also proceed by converting EG into other products, such as ethanol, methanol, or acetaldehyde, through the dehydration and hydrogenation of ethylene glycol. The intermediates from all paths can be further hydrogenated to produce a diverse range of products. The Water–Gas Shift (WGS) and hydrogenation can convert the carbon monoxide into carbon dioxide or methane.

#### 2.6.1. Gas Products

The analysis of gas product yields shows that the catalyst used has a significant impact (Table 4). The reference NiCeZr catalyst produced the highest yield of CO_2_ at 11.15%, followed by CH_4_ at 4.3% and H_2_ at 2.3%. CO and C2+ alkanes were less abundant products, with yields of 0.65% and 2.15%, respectively. The addition of Mg not only enhanced the yields of all gases but also resulted in the highest total Y_gas_ recorded for this catalyst. This was particularly evident in the performance of Y_CH4_, which experienced a significant increase to 9%. However, the modification with Cu reduces the yields of both H_2_ and CH_4_, slightly increasing those of CO and C2+. When using the Sn/NiCeZr catalyst, there was a significant reduction in the yield of all gases, with CH_4_ dropping to below 1%.

For all catalysts, the molar ratio of H_2_/CO_2_ was significantly below 1.0, which is the theoretical stoichiometric ratio for glycerol reforming (2.33). This suggests that the hydrogen produced was consumed in secondary reactions, such as the hydrogenation of liquid intermediates or the methanation of CO and CO_2_.

The hydrogen selectivity of the modified Me/NiCeZr catalysts was lower than that of the reference catalyst, despite the anticipated synergistic effects often associated with bimetallic catalyst systems [25]. It is noteworthy that the addition of Mg resulted in unexpectedly low hydrogen selectivity, despite the catalysts’ basicity promoting H_2_ production [78]. The catalyst’s high selectivity for methane suggests that the produced H_2_ was consumed in the reactor for CO/CO_2_ hydrogenation. In contrast, the catalysts modified with Cu and Sn exhibited similar hydrogen selectivity, which was associated with lower methane production. It is important to note that selectivity towards H_2_ is calculated based on hydrogenated products, while selectivity towards C compounds is calculated based on carbon. As a result, the sum of selectivities for H_2_ and all C compounds exceeds 100%.

Previous studies have reported varying effects on methane production when using different modified NiCeZr catalysts. The literature suggests that bimetallic interactions in Cu catalysts can decrease CH_4_ selectivity [79], while Sn modification may impact selectivity due to changes in Ni defect sites [80]. Additionally, Cu/NiCeZr and Sn/NiCeZr catalysts showed an increase in CO selectivity, which could indicate a subdued WGS reaction. The results are intriguing as Cu-modified catalysts are typically known to promote the WGS reaction [37]. It is possible that the high density of acid sites on both catalysts could hinder their WGS activity [74]. This is supported by the increase in selectivity for C2+ alkanes for both catalysts.

#### 2.6.2. Liquid Products

**Figure 7:**
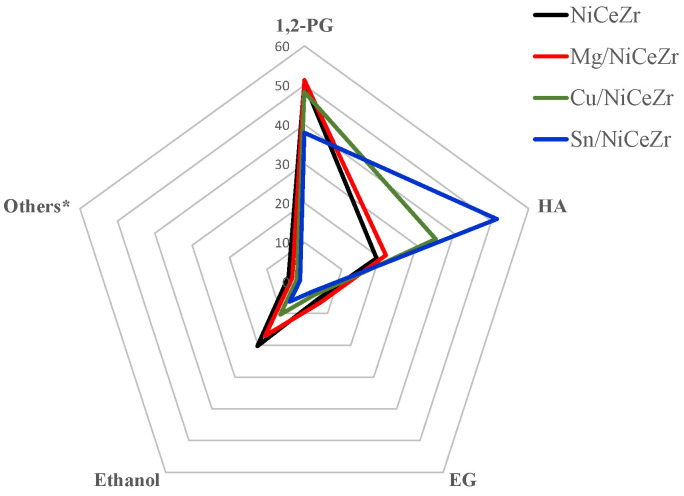
Selectivity towards primary liquid products. * Others include methanol, acetone, and 1-propanol.

As illustrated in Figure 6, a significant portion of the carbon feed is acquired in liquid form. Figure 7 displays the selectivity towards primary liquid products. The primary liquid products obtained were 1,2-PG, HA, ethanol, and EG, which accounted for over 95% of the total. Other minor products included methanol, acetone, and 1-propanol. The highest selectivity was found for 1,2-PG, with no notable differences among them (around 50%), except for Sn/NiCeZr catalysts. However, the selectivity to 1,2-PG of Sn/NiCeZr decreased to 37.9%, at the expense of an increase in selectivity for HA. The formation of 1,2-PG (though the hydrogenation of HA) may be suppressed due to the lower density of metal sites in the catalyst. The selectivity towards HA was increased by the bimetallic Cu/NiCeZr and Sn/NiCeZr catalysts, likely due to their higher acidity that enhances HA formation by dehydrating glycerol to HA. However, the Mg/NiCeZr catalyst, which potentially has a more electronegative Ni, showed lower dehydration activity, resulting in reduced HA selectivity (21.7%). The dehydration of glycerol produces HA, which is then hydrogenated to form 1,2-PG (Scheme 1). The difference in product distribution suggests that the metallic sites in NiCeZr and Mg/NiCeZr had a higher hydrogenation capacity.

There was a balance between the production of EG and ethanol, both products from the dehydrogenation path in Scheme 1. The formation of ethanol through the dehydration and subsequent hydrogenation of EG suggests enhanced hydrogenation activity at the metallic sites of NiCeZr and NiCeZr-Mg catalysts. This highlights the bifunctional nature of these catalysts, where a synergistic interplay between acid and metallic sites plays a crucial role in substrate transformation.

The liquid products showed selectivity towards the HA and 1,2-PG pathway, rather than the EG and ethanol pathway (Table 5). The promotion with Cu and Sn further enhanced this preference, which is consistent with their higher acid sites’ density.

For all catalysts studied, primary products (single C–O or C–C bond scission) were more prevalent than secondary products, with selectivity for primary products exceeding 75%. The undoped catalyst exhibited the highest selectivity for secondary products, while for the Cu- and Sn-doped catalysts, it decreased significantly, due to their very low metal availability.

The analysis of selectivity focused on the breakage of C–C and C–O bonds and revealed a predominance of C–O scission, with selectivity surpassing 92% (Table 5). The Cu/NiCeZr and Sn/NiCeZr catalysts exhibited the highest selectivity for C–O bond cleavage. The acidic sites had a significant impact on the competitive nature of C–C and C–O bond cleavages. In summary, the liquid product had a lower O/C ratio than the feedstock, indicating a lower oxygen content in the products. The catalysts that showed higher selectivity to C–O scission also demonstrated greater hydrodeoxygenation capacity.

Table S3 (Supplementary Materials) shows a comparison between the results of our catalysts with other works in the literature regarding the production of value-added products from glycerol without external hydrogen. Our catalysts showed high glycerol conversion and selectivity towards 1,2-propylene glycol, comparable or superior to previous studies. This achievement is particularly noteworthy as it was accomplished without the addition of external hydrogen, indicating a significant advantage in terms of process efficiency and sustainability.

The undoped NiCeZr catalyst stability studies (Figure S7, Supplementary Materials) demonstrate a gradual decrease in glycerol conversion over 30 h TOS, indicating a decline in catalytic efficiency for glycerol processing. The yield for liquid products also decreased, while the yield for gas remained constant. The selectivity for 1,2-PG may vary due to the dynamic nature of the catalytic sites during the reaction or a balance between the production of gaseous and liquid products. Nickel leaching (see below) is the most likely cause of the decrease in glycerol conversion and liquid product yield, resulting in the loss of active metal sites. Overall, the evidence points to nickel leaching as the primary factor in the decrease in glycerol conversion and liquid product yield. This loss may alter the acidity of the catalyst surface, potentially affecting the selectivity towards 1,2-PG. However, the steady production of gaseous byproducts suggests that these may arise from reaction pathways that are less affected by the nickel content and acidity of the catalyst. The breakdown of stable intermediates or byproducts that are not primarily formed through nickel’s catalytic action may be the cause.

### 2.7. Characterization of Spent Catalysts

Designing a catalyst for aqueous-phase biomass treatment applications presents numerous challenges, including deactivation due to metal leaching, phase transformations, or metal sintering, among other factors [17]. To explore the changes experienced by the catalysts, spent catalysts were examined.

The N_2_ adsorption–desorption isotherms of all the spent catalysts (Figure S8, Supplementary Materials) were of type IV, as for the reduced assays. However, the isotherms of the base NiCeZr and Mg/NiCeZr spent catalysts showed a hysteresis loop closure at slightly higher relative pressures than in the reduced materials (Figure S8 in Supplementary Materials), while for the spent Cu/NiCeZr this occurred at lower relative pressures. These results suggest that the desorption of the adsorbate from the pores is more challenging, possibly because of surface modifications or pore blockages that occurred during use. The PSD for the spent reference catalysts widened, while the PSD for the Mg/NiCeZr catalysts narrowed and shifted towards lower pores. Minor changes were observed for Cu/NiCeZr and Sn/NiCeZr catalysts. The S_BET_ increased by 15–25% for all spent catalysts except for the Mg/NiCeZr catalyst, which showed a decrease. It seems that the BET surface area variation was significantly affected by the leaching of Ni and Me (see below). In summary, the texture of all catalysts changed under hydrothermal conditions. Cu and Sn were found to be the promoters that best preserved the textural properties.

**Figure 8:**
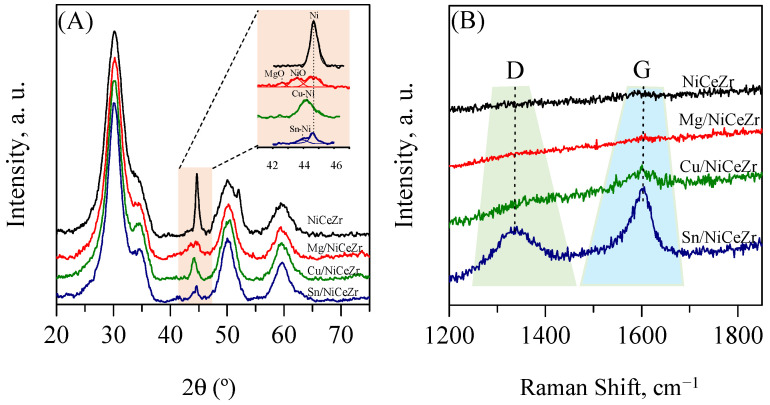
(**A**) XRD patterns and (**B**) Raman spectra of used catalysts.

The XRD profiles of the spent catalysts (Figure 8A) were similar to their respective reduced forms. However, upon closer inspection, it was discovered that there were changes in the peaks’ intensity and width when compared to their reduced forms. For example, the intensity of the Ni^0^ peak noticeably increased for the reference NiCeZr after usage, and a new peak at 51.8° emerged. Both features indicate an enlargement in the crystal size of metallic nickel. The spent Mg/NiCeZr catalyst showed a new peak from MgO (ICDD #00-045-0946), accompanied by a decrease in the intensity of the Ni^0^ peak, indicating a reduction in the crystal size of metallic nickel. The XRD profile of the spent Cu/NiCeZr catalyst revealed the absence of the Ni^0^ peak, leaving only the peak corresponding to the Ni-Cu alloy. In the case of Sn/NiCeZr, the intensity of the Ni^0^ peak slightly decreased. The study showed that the dopants were effective in preserving the metallic nickel at the core of the catalyst, despite potential surface oxidation [76]. The size of the nickel crystallites doubled in the spent NiCeZr catalyst compared to its reduced form and showed little variation for the Mg- and Sn-doped catalysts (Table 6). For Cu/NiCeZr catalysts, the size increased by 50%. These results confirm that the impregnation of Mg and Sn effectively prevented nickel coalescence during the reaction. Despite these changes in the size of the metallic nickel, the main XRD feature for the ceria–zirconia solid solution remained consistent, indicating no phase variation.

The Raman analysis conducted on the carbon region of the catalysts after the reaction (Figure 8B) revealed the presence of D and G bands, specifically for the Sn/NiCeZr catalyst. These bands indicate the formation of carbon-based residues that were not detected with the XRD analysis. The D band signals amorphous carbon types, whereas the G band points to graphitic forms [81]. The low intensity of these bands suggests a minimal presence of carbon deposits, except for the Sn-doped assay, which has the lowest oxygen vacancies and highest acidity. This reduced accumulation of carbon was attributed to the role of oxygen vacancies within the catalyst, which helps to mitigate carbon deposition [82].

The leaching of the active phase can cause irreversible catalyst deactivation, which is a common problem in hydrothermal environments. The NiCeZr catalyst showed the highest Ni and Ce leaching, which notably decreased upon doping, specifically with Cu and Sn. Among the dopants, only Mg experienced leaching, at values higher than 40%. The detachment of Mg could be promoted by the formation and subsequent elution of Mg organometallic complexes. Zr was revealed to be the most resistant to leaching. These results show that the dopants can help stabilize nickel particles under harsh hydrothermal conditions.

Accordingly, an in situ treatment with H_2_ could remove the coke and reduce the Ni surface that has been oxidized in the operation. However, the reduction treatment could only partially regenerate the catalyst due to the irreversible catalyst deactivation caused by metal leaching. Alternative strategies that aim to stabilize the metal nanoparticles against leaching could hinder catalyst deactivation.

## 3. Materials and Methods

### 3.1. Materials

#### 3.1.1. Chemicals

The catalysts were synthesized using the following reagents: cerium(IV) nitrate hexahydrate (Ce(NO_3_)_3_·6H_2_O, M_n_ = 434.22, 99%), zirconium(IV) nitrate hydrate (ZrO(NO_3_)_2_·xH_2_O, M_n_ = 231.23 anhydrous basis, 99%), nickel nitrate hexahydrate (Ni(NO_3_)_2_·6H_2_O, M_n_ = 290.79, 99.99%), cetyltrimethylammonium bromide (CTAB) (C_16_H_33_N(CH_3_)_3_Br, M_n_ = 364.45, 99%), sodium hydroxide (NaOH, 98%), magnesium nitrate hexahydrate (Mg(NO_3_)_2_·6H_2_O, M_n_ = 256.4, 99.999%), copper(II) nitrate trihydrate (Cu(NO_3_)_2_·3H_2_O, M_n_ = 187.6 anhydrous basis, 99-104%), purchased from Sigma-Aldrich (Saint Louis, MO, USA), tributyltin acetate (C_14_H_30_O_2_Sn, M_n_ = 349.1, 97.0%), purchased from Merck (Darmstadt, Germany), isopropyl alcohol ((CH_3_)_2_CHOH, M_n_ = 60.10, 99.8%), purchased from PanReac (Castellar del Vallés, Spain), and deionized water.

#### 3.1.2. Synthesis of Catalysts

The procedure for synthesizing the base NiCeZr catalyst (10 wt.%Ni, Ce/Zr = 15/85 at./at.) can be found elsewhere [26]. In brief, it was synthesized using a one-pot co-precipitation method with cetyltrimethylammonium bromide (CTAB) as a surfactant, and calcined for 4 h at 500 °C (heating ramp of 1 °C/min). The modifiers were added to NiCeZr using a wet impregnation method with aqueous solutions (50:50 isopropanol:water mixture for Sn) of the corresponding metal salt (at 1 wt.% concentration). The solid product was dried at 100 °C overnight and calcined at 500 °C for 4 h using a heating ramp of 1 °C/min. The modified solids were named Me/NiCeZr (Me = Mg, Cu or Sn).

### 3.2. Characterization of the Catalysts

The chemical composition of the catalysts was analyzed using inductively coupled plasma atomic emission spectroscopy (ICP-AES) (7700, Agilent, Santa Clara, CA, USA), following a standard acid digestion procedure. To quantify the metals leached into the final liquid product, inductively coupled plasma mass spectrometry (ICP-MS) (XSeries 2, Thermo Scientific, Waltham, MA, USA) was employed. The textural properties of the solids were evaluated by N_2_ adsorption–desorption isotherms at 77 K (TRISTAR II 3020, Micromeritics, Norcross, GA, USA). Before adsorption measurements, the samples were outgassed at 400 °C for 10 h to eliminate moisture and adsorbed gases. The BET method was used to determine the specific surface area, while the BJH (adsorption-branch) method was used to obtain the average pore size.

The powder X-ray diffraction (XRD) patterns of the solids were obtained using a PANalytical X’Pert-Pro instrument, covering a range of 5 to 90° 2θ with a step size of 0.026° and a duration of 598 s, and employing monochromatized CuKα radiation (λ = 1.5418 Å). The crystallite size was determined by applying the Scherrer equation. The identification of crystalline phases was conducted by comparing them with the ICDD database. The degree of particle agglomeration was estimated by calculating the ratio of the average particle volume, assumed to be spherical based on the S_BET_, to the crystallite volume obtained from the XRD results.

The Raman spectra were recorded using a Renishaw InVia Raman spectrometer coupled with a Leica DMLM microscope. The analysis employed a 514 nm ion argon laser (Modu-Laser) and a holographic grating of 1800 lines/mm. Each spectrum was acquired over a 20 s period with 10 accumulations, ensuring the laser power remained consistently below 2 mW. The measurements were conducted within a spectral window of 150–1500 cm^−1^.

The reducibility of the calcined solids was evaluated through a temperature-programmed reduction (H_2_-TPR) technique using a Micromeritics AutoChem 2920 apparatus. The samples were initially cleaned by purging with He at a flow rate of 50 mL/min at 500 °C for 30 min, followed by cooling to 40 °C. The H_2_-TPR analysis involved flowing a 5% H_2_/Ar gas mixture over the samples and incrementally increasing the temperature from room temperature to 900 °C at a rate of 10 °C/min. The Thermal Conductivity Detector (TCD) continuously monitored the outflow from the reactor, while the resulting water was trapped.

The oxygen storage capacity (OSC) was evaluated using a Micromeritics AutoChem 2920 instrument connected to a Pfeiffer Vacuum OmniStar mass spectrometer. After surface cleaning by heating at 500 °C in He flow for 30 min, the catalyst was reduced for an hour at 600 °C in a 5% H_2_/Ar flow. Then, the gas flow was switched to He and the sample cooled down to 235 °C. At this temperature, the samples experienced ten pulses of oxygen 5% O_2_/He, followed by ten pulses of 5% CO/He. The OSC was determined as the sum of the released oxygen moles after each CO pulse.

The metallic surface area was further characterized using static H_2_ chemisorption and analyzed using a Micromeritics ASAP 2020 instrument. Initially, the sample was subjected to surface cleaning under He flow at 350 °C to remove any adsorbed species. Subsequently, the temperature was reduced to 50 °C, followed by H_2_ reduction at 600 °C with a flow rate of 50 mL/min for 1 h. To eliminate any residual H_2_, the sample was then degassed for 1 h. The chemisorption process involved a double H_2_ adsorption isotherm conducted at 5 °C, incorporating an intermediate degassing step. This temperature was specifically chosen to reduce the occurrence of H_2_ spillover. The quantitative assessment of the chemisorbed hydrogen was determined by the difference between the first and second isotherms, providing insights into the metallic surface, metal sites, and dispersion.

The acidity of the catalysts was assessed by ammonia chemisorption followed by temperature-programmed desorption (NH_3_-TPD) (AutoChem 2920, Micromeritics, Norcross, GA, USA). The catalyst’s surface was initially cleaned by heating it to 500 °C under a stream of He for 30 min. Following this, the catalyst was reduced at 600 °C for an hour in a 5% H_2_/Ar stream, and then cooled to 90 °C under a flow of He. Then, six pulses of 10% NH_3_/He (loop volume 0.5312 mL) were introduced to quantify the chemisorbed ammonia. Subsequently, He was flowed for one hour to evacuate the system. Then, the sample’s temperature was gradually increased to 900 °C at a rate of 10 °C/min, following the reactor exhaust using the TCD. The number of acid sites was determined by integrating the areas of the NH_3_ pulses, while the acid strength was inferred from the desorption temperatures.

The oxidation states of the surface metals in both the calcined and reduced forms of the catalysts were investigated using X-ray photoelectron spectroscopy (XPS). The analysis was conducted on a SPECS spectrometer, which is equipped with a Phoibos 150 1DDLD analyzer and utilizes monochromatic Al Kα X-ray sources (1486.7 eV). The pass energy was finely tuned in increments of 0.05 eV to 30 eV. For samples requiring in situ reduction, the procedure was carried out at 600 °C for 1 h. Spectrometer calibration was achieved using the Ag 3d_5/2_ peak at 368.26 eV, and the binding energy was referenced to the C 1s peak of adventitious carbon at 284.6 eV. Peak deconvolution was performed after Shirley background subtraction, employing a combined Gaussian–Lorentzian function. This analysis was facilitated by the use of CASA XPS software, ensuring accurate interpretation of the data.

### 3.3. Catalytic Tests

Catalytic testing for glycerol hydrogenolysis was conducted in a bench-scale fixed-bed upflow reactor (Microactivity Effi, PID Eng&Tech, Alcobendas, Spain) using a 10 wt.% glycerol (99.5%, PanReac, Castellar del Vallés, Spain) aqueous solution. The Electronic Supplementary Information includes a detailed schematic representation of the experimental setup (Figure S9). The reaction conditions were maintained at 235 °C and 35 bar, with a WHSV of 12 h^−1^. The catalyst underwent an in situ reduction at 600 °C for 1 h under a 10% H_2_/He flow before the experiments. The total on-stream time (TOS) for the reaction was 3 h. Product separation into gaseous and liquid phases was achieved using a Peltier device. The gaseous output was continuously swept with a He flow of 40 mL/min after the backpressure regulator and analyzed online via microGC-TCD (Agilent 490), equipped with Al_2_O_3_-KCl, PPQ, and MS5A columns using He as a carrier, and MS5A column utilizing Ar. The liquid samples, collected hourly, were analyzed off-line using a GC-FID system (Agilent 6890N with a DB-Heavy Wax column). The carbon content in the liquid phase was determined using a Shimadzu TOC-L apparatus, ensuring a carbon balance exceeding 90% in all experiments.

### 3.4. Equations Used for Calculations for Catalytic Results

Glycerol conversion (X_gly_) was determined using Equation (1):

(1) $$X_{gly}(\%)=100\times \frac{F_{gly}^{in}-F_{gly}^{out}}{F_{gly}^{in}}$$

where $F_{gly}^{in}$ and $F_{gly}^{out}$ are the molar flow of glycerol at the reactor inlet and outlet, respectively. The following methodology was used to calculate gas and liquid product yields based on carbon content (Equations (2) and (3)):

(2) $$Y_{gas}(\%)=100\times \frac{F_{C,gas}^{out}}{3\times F_{gly}^{in}}$$

(3) $$Y_{liq}(\%)=100\times \frac{F_{C,liq}^{out}}{{3\times F}_{gly}^{in}}$$

$F_{C,gas}^{out}$ and $F_{C,liq}^{out}$ are the total molar flow of carbon in the outlet gas and liquid stream, respectively (excluding glycerol). These metrics range between 0 and 100%.

For the carbon-containing products, selectivity (S_i_) and yield (Y_i_) for product i were determined based on its carbon content, as follows (Equations (4) and (5)):

(4) $$S_{i}\left(\%\right)=100\times \frac{F_{C.i}^{out}}{\sum_{i=1}^{n}F_{C,i}^{out}}$$

(5) $$Y_{i}\left(\%\right)=100\times \frac{F_{C.i}^{out}}{{3\times F}_{gly}^{in}}$$

where $F_{C,i}^{out}$ is the carbon flow in the product i. These metrics range from 0% (no production of C-containing i) to 100% (only i is obtained as a C-containing product).

The selectivity to hydrogen was calculated as the percentage of H_2_ in relation to the total hydrogen released in all gaseous products (Equation (6)).

(6) $$S_{H_{2}}\left(\%\right)=100\times \frac{{2\times F}_{H_{2}}^{out}}{F_{H,gas}^{out}}$$

The hydrogen yield (Y_H2_) represented the ratio of produced H_2_ to the ideally produced by glycerol APR (Equation (7)):

(7) $$Y_{H_{2}}\left(\%\right)=100\times \frac{1}{7}\times \frac{F_{H_{2}}^{out}}{F_{gly}^{in}}$$

where $F_{H_{2}}^{out}$ and where $F_{H,gas}^{out}$ are the H_2_ and total H flow in the outlet gas, respectively. These metrics range from 0% (no H_2_ exhaust) to 100% (H_2_ being the only H-containing molecules in the gas phase).

## 4. Conclusions

The investigation aimed to improve the applicability of NiCeZr catalyst doping at the surface level with Mg, Cu, and Sn, for the sustainable valorization of biomass through glycerol hydrogenolysis. Modifications slightly expanded pore sizes for Cu- and Sn-doped catalysts, but all doped variants experienced a decrease in surface area, particularly for Cu/NiCeZr. The redox behavior and oxygen storage analyses demonstrated the distinct effects of each dopant. Mg had a slight impeding effect on the reduction temperatures of the catalysts, while Cu facilitated and Sn increased the temperatures. The structural analyses showed the impact of dopants on catalyst properties. This also influenced the redox properties and oxygen vacancies of the catalysts.

The efficiency and selectivity of glycerol conversion were significantly impacted by the modifications. It was observed that dopants had a significant effect on the efficiency and selectivity of glycerol conversion in NiCeZr catalysts. The undoped catalyst had a conversion rate of 75.3%, with a selectivity rate of 93.5% for the C–O bond cleavage. However, after doping, the conversion rate slightly decreased, and the selectivity for cleaving C–O bonds increased for the Cu and Sn variants. Furthermore, the introduction of dopants led to an increased selectivity for cleaving C–C bonds. These results can be correlated with the density of acid and metal sites, as well as oxygen vacancies.

In post-reaction assessments, the spent catalysts were found to be structurally and functionally stable. The dopants were effective in preserving the metallic nickel core and mitigating sintering effects. However, the Mg/NiCeZr catalyst exhibits significant Mg leaching, which needs to be addressed for further optimization. The findings show the complex connection between changes in structure and catalytic performance, providing useful insights for creating effective and durable catalysts for biomass valorization.

## Figures and Tables

**Table 1:** Chemical composition of the bulk, crystallite sizes, lattice parameter, and available metal sites.

Catalyst	Me (wt.%)	Ni (wt.%)	Ce/Zr (at./at.)	S_BET_ (m^2^/g)	d_NiCeZr.XRD_ (nm)	DA ^a^	Lattice * (Å)	d_Ni_^0^ (nm)	Ni^0^ Sites (×10^−19^) (Sites/g_cat_) ^b^
NiCeZr	0	9.2	14.1/85.9	235 (190)	2.7 (2.9)	1.6	a = 3.6094 (3.6559) c = 5.2539 (5.2705)	11.4	4.2
Mg/NiCeZr	1.05	9.2	13.6/86.4	226 (197)	2.8 (3.3)	1.6	a = 3.6241 (3.6615) c = 5.2315 (5.2181)	10.9	2.9
Cu/NiCeZr	1.06	9.2	15.7/84.3	224 (202)	2.3 (2.9)	1.9	a = 3.6452 (3.6698) c = 5.2014 (5.2232)	5.0	0.7
Sn/NiCeZr	0.82	9.2	14.6/85.4	200 (199)	2.5 (3.0)	2.0	a = 3.6366 (3.6720) b = 5.2244 (5.2239)	20.1	0.5

* Calculated as tetragonal: $\frac{1}{d^{2}}=\frac{\left(h^{2}+k^{2}\right)}{a^{2}}+\frac{l^{2}}{c^{2}}$ (a = b ≠ c). Values in parenthesis correspond to the reduced forms. ^a^ DA: degree of agglomeration, calculated as d_BET_/d_XRD_. ^b^ calculated from H_2_ chemisorption.

**Table 2:** Redox and surface characteristics of the materials.

Catalyst	H_2_ Uptake (mmolH_2_/g)				OSC (mmolo_/_g_cat_)	Sites Density (Sites/nm^2^)		Metal/Acid (Sites/Sites)
	Overall ^a^	Peak				Metal ^b^	Acid ^c^	
		α	β	γ				
NiCeZr	2.20 (106)	0.11	0.44	1.65	63	0.22	0.36	0.62
Mg/NiCeZr	1.62 (78)	0.03	0.25	1.34	60	0.15	0.30	0.49
Cu/NiCeZr	2.28 (101)	0.10	0.62	1.56	65	0.03	0.42	0.08
Sn/NiCeZr	2.28 (103)	0.04	0.46	1.78	43	0.02	0.51	0.05

In parenthesis, the percentage of reduction with respect to the theoretical ratio for the complete reduction of all nickel, cerium, and Me ions, assuming they are present as Ni^2+^, Ce^4+^, Cu^2+^, and Sn^4+^, respectively. ^a^ Uptake up to 900 °C. ^b^ from H_2_ chemisorption. ^c^ from ammonia chemisorption.

**Table 3:** Surface chemical composition from the XPS analysis.

Catalyst	Ce/Zr	Ni/(Ce + Zr)	Ce^3+^/(Ce^4+^ + Ce^3+^)	Bulk Me/ (Ni + Ce + Zr)	Me/(Ni + Ce + Zr)	Ni^0^/Ni_Total_
NiCeZr	0.13 (0.17)	0.17 (0.08)	0.16 (1.0)	0	0 (0)	0.0 (0.70)
Mg/NiCeZr	0.18 (0.21)	0.33 (0.10)	0.27 (1.0)	0.05	0.11 (0.10)	0.0 (0.79)
Cu/NiCeZr	0.17 (0.22)	0.39 (0.10)	0.19 (1.0)	0.02	0.06 (0.05)	0.0 (0.89)
Sn/NiCeZr	0.16 (0.23)	0.31 (0.10)	0.19 (1.0)	0.01	0.03 (0.02)	0.0 (0.86)

In parenthesis, values for solids in situ reduced at 600 °C for 1 h in 10% H_2_/He flow.

**Table 4:** Gas product yields and selectivities.

Catalyst	Yield (%)					Selectivity (%)					H_2_/CO_2_
	H_2_	CH_4_	CO	CO_2_	C2+	H_2_	CH_4_	CO	CO_2_	C2+	
NiCeZr	2.3	4.30	0.65	11.15	2.15	32.2	23.5	3.6	61.1	11.8	0.48
Mg/NiCeZr	3.2	9.21	0.76	17.55	3.30	24.8	29.9	2.5	56.9	10.7	0.43
Cu/NiCeZr	1.8	2.46	0.99	8.07	3.01	32.5	16.9	6.8	55.5	20.8	0.51
Sn/NiCeZr	0.6	0.69	0.43	1.60	1.61	29.5	16.0	10.1	36.9	37.0	0.87

**Table 5:** Selectivities to primary and secondary products, type of cleaved bond, and yield to 1,2-PG (in %).

Catalyst	Primary Products	Secondary Products	C–C	C–O	O/C	Y_1,2-PG_
NiCeZr	75.5	24.5	6.6	93.4	0.65	28.5
Mg/NiCeZr	79.6	20.4	7.9	92.1	0.66	19.3
Cu/NiCeZr	87.9	12.1	5.1	94.9	0.66	25.0
Sn/NiCeZr	92.6	7.4	3.9	96.1	0.67	16.7

Primary products: single C–O or C–C bond scission (EG, 1,2-PG, HA). Secondary products: more than a single C–O or C–C bond scission (ethanol, methanol, acetone, 1-propanol).

**Table 6:** Textural properties, crystallite size, and concentration of the leached metals of Me/NiCeZr catalysts after 3 h of use.

Catalyst	S_BET_ (m^2^/g)	ΔV_pore_ (%)	Δd_pore_ (%)	d_Niº_ (nm)	Leaching (wt.%)			
					Ni	Me	Ce	Zr
NiCeZr	218 (+15%)	+21	+7	21.0	3.9	0.0	0.9	0.0
Mg/NiCeZr	211 (−7%)	−21	−15	8.9	2.1	40.3	0.1	0.0
Cu/NiCeZr	279 (+25%)	+28	+16	7.8	0.9	0.0	0.1	0.0
Sn/NiCeZr	246 (+23%)	+35	−7	18.2	0.6	0.0	0.0	0.0

In parenthesis, percentage variation with respect to freshly reduced forms.

## Data Availability

The raw data supporting the conclusions of this article will be made available by the authors on request.

## Supplementary Materials

The following supporting information can be downloaded at: https://www.mdpi.com/article/10.3390/ijms25063484/s1. References [83,84,85,86,87,88,89,90] are cited in the Supplementary Materials.
