# Supplementary material for: Hydrogenolysis of Glycerol over NiCeZr Catalyst Modified with Mg, Cu, and Sn at the Surface Level
Source: Int J Mol Sci. 2024 Mar 20;25(6):3484. doi: 10.3390/ijms25063484 (PMC10970669; doi:10.3390/ijms25063484)
Supplement: Supplementary file 1 [file ijms-25-03484-s001.zip › ijms-2911990-supplementary.pdf]

Supplementary Material

Hydrogenolysis of Glycerol over NiCeZr Catalyst Modified with Mg, Cu, and Sn at the Surface Level

Norberto Vera-Hincapie [1], Unai Iriarte-Velasco [2], Jose Luis Ayastuy [1,*] and Miguel Ángel Gutiérrez-Ortiz [1]

[1] Department of Chemical Engineering, Faculty of Science and Technology, University of the Basque Country UPV/EHU, Sarriena S/N, 48940 Leioa, Spain; norberto.vera@ehu.eus (N.V.-H.); miguelangel.gutierrez@ehu.eus (M.Á.G.-O.)

[2] Department of Chemical Engineering, Faculty of Pharmacy, University of the Basque Country UPV/EHU, Paseo de la Universidad, 7, 01006 Vitoria, Spain; unai.iriarte@ehu.eus

* Correspondence: joseluis.ayastuy@ehu.eus

1. Characteristics of the Pores

Table S1. Pore volume and average pore sizes.

Catalyst	V_{pore} (cm³/g)	d_{pore} (nm)
NiCeZr	0.270/0.252/0.306	4.2/4.2/4.5
Mg/NiCeZr	0.293/0.272/0.215	4.1/4.6/3.9
Cu/NiCeZr	0.309/0.275/0.352	4.3/4.5/5.2
Sn/NiCeZr	0.242/0.247/0.333	3.7/4.2/3.9

Calcined/reduced/spent. n.a.: not analyzed.

2. N_2 Isotherms and PSD of the Calcined and Reduced Solids

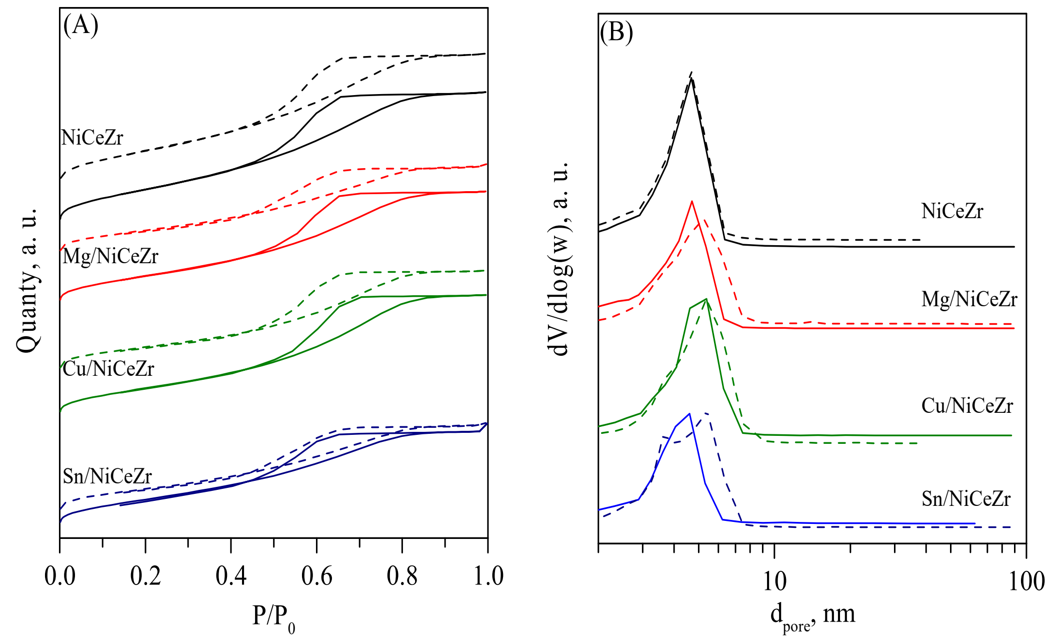

Figure S1. (**A**) Nitrogen adsorption-desorption isotherms of Me/NiCeZr solids, and (**B**) pore size distribution. Calcined solids (solid lines) and reduced solids (dashed lines).

3. NH_3-TPD and Acid Strength Distribution

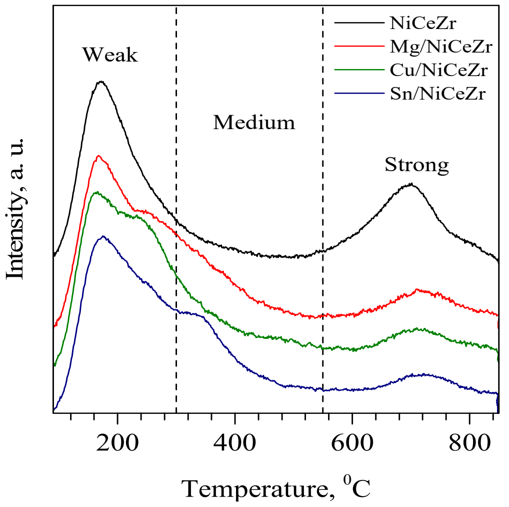

Figure S2. NH_3-TPD profiles for the Me/NiCeZr solids.

Table S2. Distribution of the strength of acidity by NH_3-TPD on reduced catalysts.

Catalyst	Acid strength distribution (%)		
	**Weak**	**Intermediate**	**Strong**
NiCeZr	68	2	30
Mg/NiCeZr	73	10	17
Cu/NiCeZr	70	11	19
Sn/NiCeZr	71	12	17

4. XPS

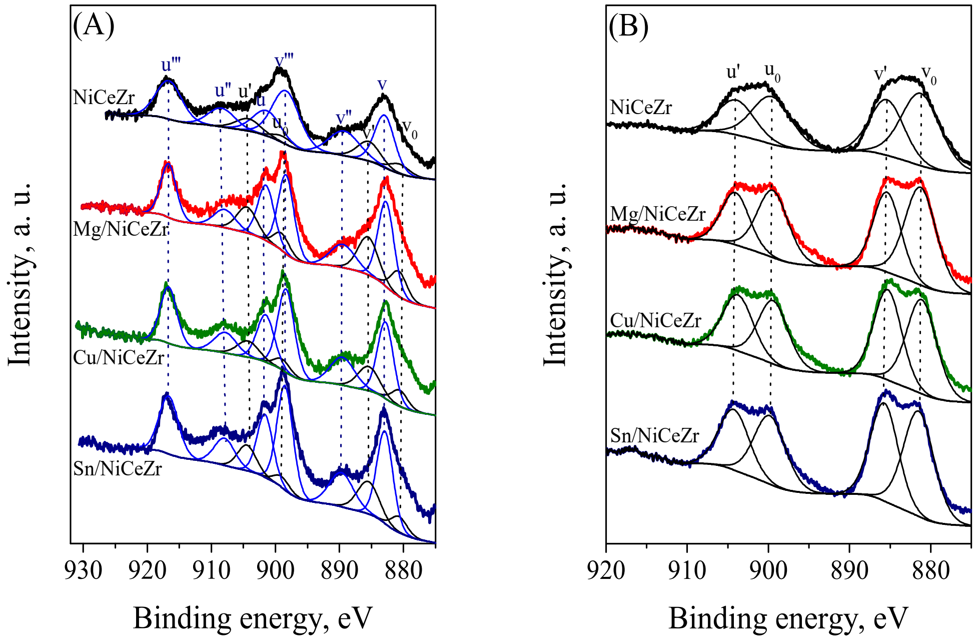

Figure S3. Detailed Ce 3d spectra for the calcined (**A**) and reduced (**B**) solids.

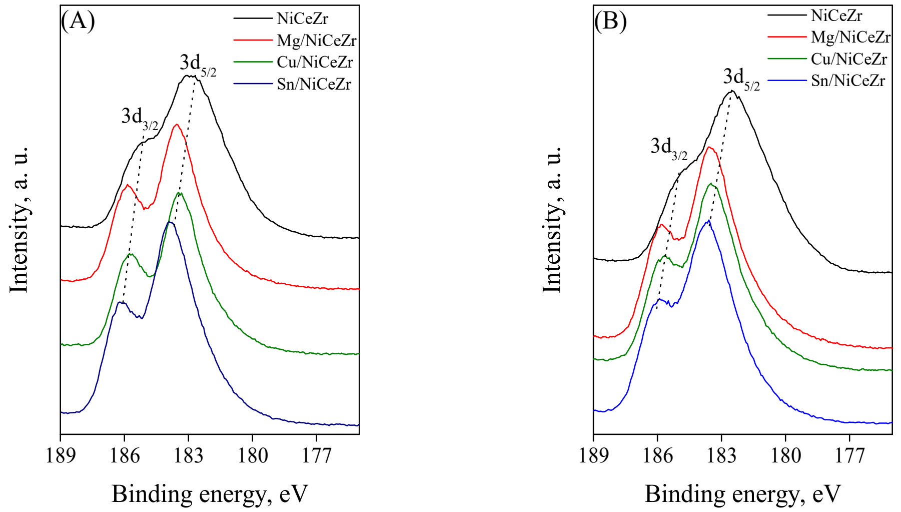

Figure S4. Detailed Zr 3d spectra of calcined (**A**) and reduced (**B**) solids. Peaks assignation in the manuscript.

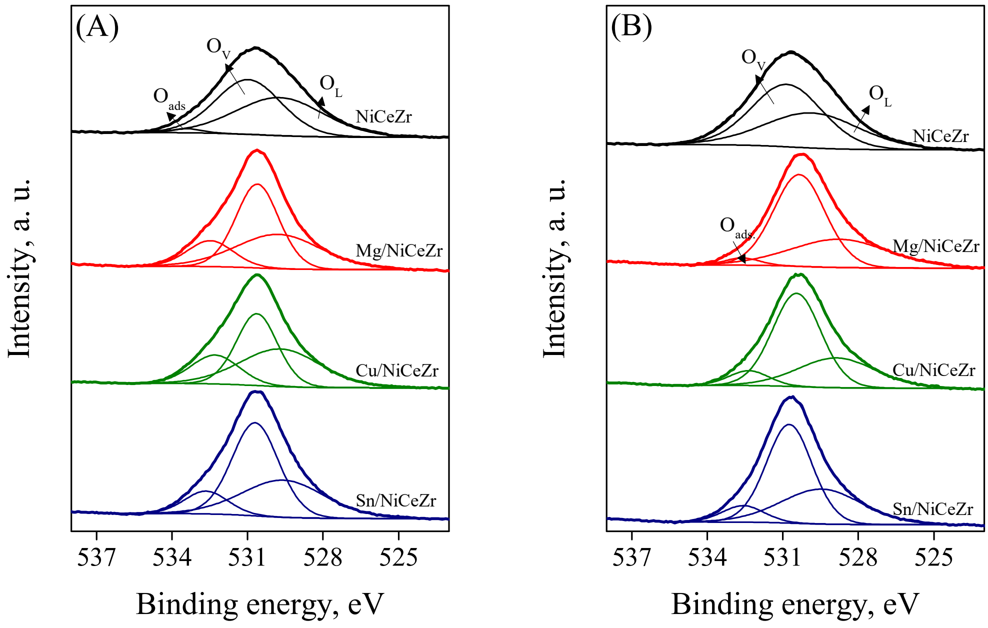

Figure S5. Detailed O 1s spectra of fresh (**A**) and reduced (**B**) solids.

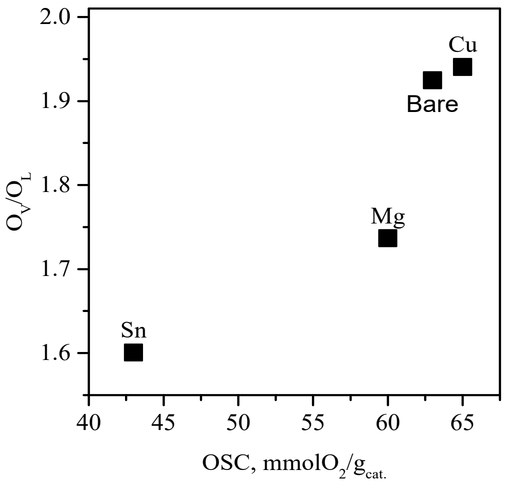

Figure S6. Correlation between OSC and O_V/O_L ratio.

5. Reaction Results

Table S3. State of the art of Ni-based catalysts for the glycerol hydrogenolysis without external hydrogen.

Catalyst	Reactor	Conditions T (°C)/ P (bar)/TOS (h)	Glycerol concentration (wt.%)	H_2 source	X_{Gly} (%)	$S_{1,2\,PG}$ (%)	Ref.
Raney nickel	Batch	190/10/8	8	External	32.0	79.0	[1]
Ni/AC	Batch	200/50/6	25	External	7.4	18.3	[2]
Ni/SiO_2	Batch	280/10/8	5.75	External	30.0	98.0	[3]
Ni/CeO_2	Batch	230/20 (N_2)/1	10	In-situ	54.3	15.3	[4]
CuNi/ZSM-5	Tubular	250/40 (N_2)/6	10	In-situ	87.0	31.0	[5]
Ni/Al-Fe	Tubular	227/34/3	10	In-situ	42.3	65.0	[6]
Ni/Cu/ZrO_2	Tubular	230/35/1	5	In-situ	74.3	69.4	[7]
28Ni/Al_3Fe_1	Tubular	227/34/3	10	In-situ	42.3	51.0	[6]
MoNiAl	Tubular	235/45/4	10	In-situ	77.0	33.0	[8]
NiCeZr	Tubular	235/35/4	10	In-situ	75.3	52.8	Present work
Mg/NiCeZr	Tubular	235/35/4	10	In-situ	71.3	51.3	Present work
Cu/NiCeZr	Tubular	235/35/4	10	In-situ	64.7	48.3	Present work
Sn/NiCeZr	Tubular	235/35/4	10	In-situ	54.9	37.9	Present work

See references at the end of this file.

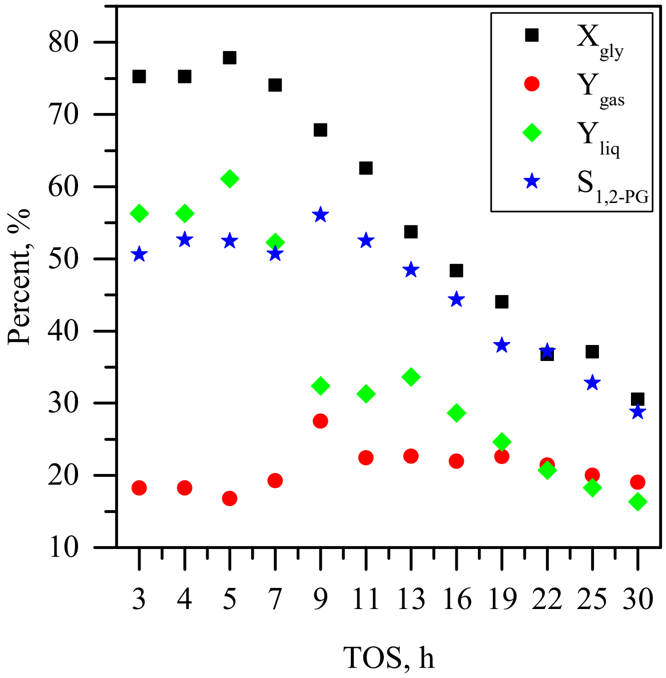

Figure S7. Reaction indices variation with TOS. Reaction conditions (10 wt.% glycerol, 235 °C and 35 bar, with a WHSV of 12 h^{-1}).

6. N_2 Isotherms and PSD of the Spent Solids

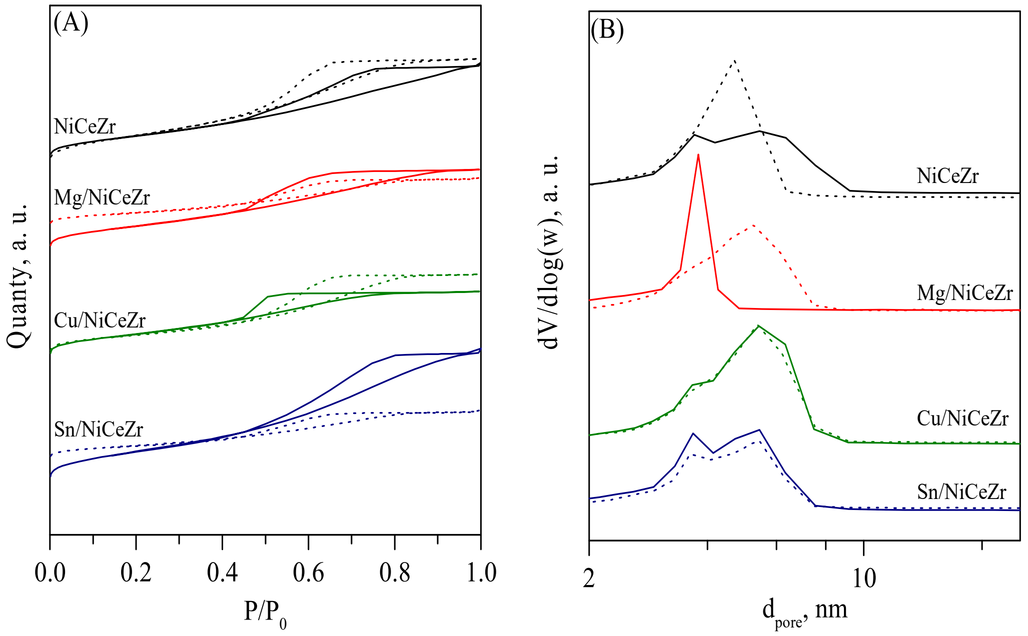

Figure S8. (**A**) Nitrogen adsorption-desorption isotherms, and (**B**) pore size distribution of Me/NiCeZr catalysts. Fresh reduced (dashed lines) and spent catalysts (solid lines).

7. Schematic Diagram of the Experimental Setup

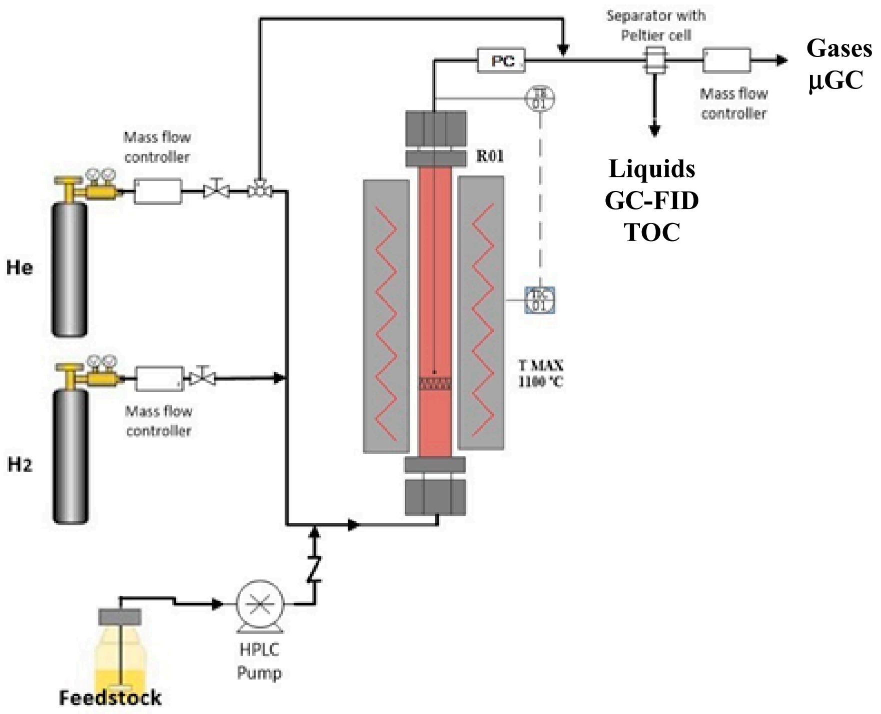

Figure S9. Schematic diagram of the experimental setup.

References

1. Perosa, A.; Tundo, P. Selective Hydrogenolysis of Glycerol with Raney Nickel. *Ind. Eng. Chem. Res.* **2005**, *44*, 8535–8537.
2. Yu, W.; Xu, J.; Ma, H.; Chen, C.; Zhao, J.; Miao, H.; Song, Q. A Remarkable Enhancement of Catalytic Activity for KBH_4 Treating the Carbothermal Reduced Ni/AC Catalyst in Glycerol Hydrogenolysis. *Catal. Commun.* **2010**, *11*, 493–497. https://doi.org/10.1016/j.catcom.2009.12.009.
3. Marinoiu, A.; Ionita, G.; Gáspár, C.L.; Cobzaru, C.; Oprea, S. Glycerol Hydrogenolysis to Propylene Glycol. *React. Kinet. Catal. Lett.* **2009**, *97*, 315–320. https://doi.org/10.1007/s11144-009-0032-2.
4. Syuhada, A.; Ameen, M.; Azizan, M.T.; Aqsha, A.; Yusoff, M.H.M.; Ramli, A.; Alnarabiji, M.S.; Sher, F. In-Situ Hydrogenolysis of Glycerol Using Hydrogen Produced via Aqueous Phase Reforming of Glycerol over Sonochemically Synthesized Nickel-Based Nano-Catalyst. *Mol. Catal.* **2021**, *514*, 111860. https://doi.org/10.1016/j.mcat.2021.111860.
5. Freitas, I.C.; Manfro, R.L.; Souza, M.M.V.M. Hydrogenolysis of Glycerol to Propylene Glycol in Continuous System without Hydrogen Addition over Cu-Ni Catalysts. *Appl. Catal. B Environ.* **2018**, *220*, 31–41. https://doi.org/10.1016/j.apcatb.2017.08.030.
6. Raso, R.; García, L.; Ruiz, J.; Oliva, M.; Arauzo, J. Aqueous Phase Hydrogenolysis of Glycerol over Ni/Al-Fe Catalysts without External Hydrogen Addition. *Appl. Catal. B Environ.* **2021**, *283*, 119598. https://doi.org/10.1016/j.apcatb.2020.119598.
7. Cai, F.; Pan, D.; Ibrahim, J.J.; Zhang, J.; Xiao, G. Hydrogenolysis of Glycerol over Supported Bimetallic Ni/Cu Catalysts with and without External Hydrogen Addition in a Fixed-Bed Flow Reactor. *Appl. Catal. A Gen.* **2018**, *564*, 172–182. https://doi.org/10.1016/j.apcata.2018.07.029.
8. Gallego-García, D.; Iriarte-Velasco, U.; Gutiérrez-Ortiz, M.A.; Ayastuy, J.L. Nickel Aluminate Spinel-Derived Catalysts for Aqueous-Phase Hydrogenolysis of Glycerol with in-Situ Hydrogen Production: Effect of Molybdenum Doping. *Appl. Catal. B Environ.* **2024**, *344*, 123671. https://doi.org/10.1016/j.apcatb.2023.123671.
